# Combining Mini-Mental State Examination and Montreal Cognitive Assessment for assessing the clinical efficacy of cholinesterase inhibitors in mild Alzheimer’s disease: a pilot study

**DOI:** 10.1007/s40520-024-02744-4

**Published:** 2024-04-17

**Authors:** Giovanna Furneri, Simone Varrasi, Claudia Savia Guerrera, Giuseppe Alessio Platania, Vittoria Torre, Francesco Maria Boccaccio, Maria Federica Testa, Federica Martelli, Alessandra Privitera, Grazia Razza, Mario Santagati, Santo Di Nuovo, Concetta Pirrone, Sabrina Castellano, Filippo Caraci, Roberto Monastero

**Affiliations:** 1 Department of Educational Sciences, University of Catania, Catania, Italy; 2 Department of Mental Health, ASP3 Catania, Alzheimer Psychogeriatric Center, Catania, Italy; 3 Department of Drug and Health Sciences, University of Catania, Catania, Italy; 4 Oasi Research Institute - IRCCS, Troina, Italy; 5 Department of Biomedicine, Neuroscience and Advanced Diagnostics, University of Palermo, Palermo, Italy

**Keywords:** Cognitive decline, Alzheimer’s Disease, MMSE, MoCA, Cholinesterase inhibitors, Psychometric tools

## Abstract

Current drugs for Alzheimer’s Disease (AD), such as cholinesterase inhibitors (ChEIs), exert only symptomatic activity. Different psychometric tools are needed to assess cognitive and non-cognitive dimensions during pharmacological treatment. In this pilot study, we monitored 33 mild-AD patients treated with ChEIs. Specifically, we evaluated the effects of 6 months (Group 1 = 17 patients) and 9 months (Group 2 = 16 patients) of ChEIs administration on cognition with the Mini-Mental State Examination (MMSE), the Montreal Cognitive Assessment (MoCA), and the Frontal Assessment Battery (FAB), while depressive symptoms were measured with the Hamilton Depression Rating Scale (HDRS). After 6 months (Group 1), a significant decrease in MoCA performance was detected. After 9 months (Group 2), a significant decrease in MMSE, MoCA, and FAB performance was observed. ChEIs did not modify depressive symptoms. Overall, our data suggest MoCA is a potentially useful tool for evaluating the effectiveness of ChEIs.

## Introduction

Currently available drugs for Alzheimer’s Disease (AD), such as cholinesterase inhibitors (ChEIs), exert only a symptomatic effect by slowing the progression of cognitive decline [1]. In this scenario, the response of AD patients to treatment with ChEIs should be measured for tailored interventions. A recent meta-analysis [2] focused on the use of the Mini-Mental State Examination (MMSE) [3] as the main tool to assess the effects of the ChEIs on cognitive decline in dementia. However, the same authors pointed out the limitations of the MMSE for its poor convergent validity and the floor/ceiling effect [2]. Accordingly, there is a need to include other psychometric instruments for assessing the clinical effectiveness of ChEIs in AD.

For instance, the Montreal Cognitive Assessment (MoCA) [4] is a global measure of cognition that has shown high sensitivity and specificity in detecting Mild Cognitive Impairment (MCI), while other specific cognitive domains and neuropsychiatric symptoms should be considered to give clearer a picture into person’s intrinsic capacity [5], in particular executive functions [6] and affective symptoms such as depression [7].

Given the importance of identifying the most sensitive tools to monitor the outcome of ChEIs treatment, the aim of this pilot study was to compare the performance of the MMSE, MoCA, Frontal Assessment Battery (FAB) [8], and Hamilton Depression Rating Scale (HDRS) [9] in assessing the effects of ChEIs effects in mild AD. Therefore, a sample of 33 patients who underwent a psychometric assessment at the beginning of pharmacological treatment (T_0_), and after 6 (Group 1) or 9 months (Group 2) of drug intake (T_1_) was enrolled.

## Materials and methods

### Participants

The study involved 33 participants with mild AD, recruited from the U.O.S. Centro Alzheimer e Psicogeriatria, ASP3, Catania, Italy. The diagnosis of probable AD was made according to National Institute on Aging and the Alzheimer’s Association guidelines [10]. Inclusion criteria regarded an adjusted MMSE score ≥ 16, and ≤ 25, while exclusion criteria concerned the presence of other neurological and/or psychiatric conditions. The entire sample was divided into two AD subgroups with different follow-up: Group 1 (17 participants) was screened before (T_0_) and after (T_1_) 6 months of drug treatment, while Group 2 (16 participants) was screened before (T_0_) and after (T_1_) 9 months of drug treatment (Table 1).

**Table 1:** Demographic characteristics of samples

	No.	Age (Mean ± SD)	Years of Education (Mean ± SD)	Sex	
**Group 1**	17	76.29 ± 6.87	7.29 ± 3.39	*Females*	14
				*Males*	3
**Group 2**	16	76.38 ± 5.965	9.19 ± 5.72	*Females*	11
				*Males*	5

### Procedure

The study was conducted in accordance with the Declaration of Helsinki and guidelines set by the Ethical Council of AIP (Italian Association of Psychology). The protocol was approved by the Internal Ethics Review Board of the Department of Educational Sciences (Section of Psychology) of the University of Catania (Ierb-Edunict-2023.05.23/02). After signing an informed consent, each participant was tested by an expert neuropsychologist twice (T_0_ and T_1_) in a single session of about 20 min each. All patients were treated with ChEIs (donepezil 10 mg/die or rivastigmine patch 9.5 mg/die) for at least 6 (Group 1) and 9 (Group 2) months.

### Measures

The psychometric protocol consisted of: (1) the Italian version of the MMSE [3]; (2) the Italian version of the MoCA [4]; (3) the Italian version of the FAB [8], a global measure to evaluate executive functioning; and (4) the Italian version of the HDRS [9] for the assessment of depressive symptoms.

### Data analysis

Given the sample size and the distribution of variables, statistical analysis was carried out using non-parametric tests with SPSS version 27 (IBM). The Mann-Whitney U test for independent samples and Wilcoxon W test for paired data were used. Performance between baseline (T_0_) and follow-up (T_1_) at 6 months (Group 1) or 9 months (Group 2) during ChEIs treatment was compared. For all analyses, significance level was set at *p* < 0.05 with related effect-sizes.

## Results

Participants of Group 1 and Group 2 did not differ at T_0_ in MMSE (U = 126; *p* = 0.73; r_g_ = 0.07), MoCA (U = 132; *p* = 0.9; r_g_ = 0.02), FAB (U = 129; *p* = 0.81; r_g_ = 0.05), and HDRS (U = 126; *p* = 0.73; r_g_ = 0.07), nor in years of education (U = 119; *p* = 0.51; r_g_ = 0.12).

Firstly, the effect of ChEIs treatment on cognitive performance and depressive symptoms in Group 1 after 6 months was evaluated (Table 2). The comparison between T_0_ and T_1_ showed no statistically significant differences in MMSE scores. Similarly, no statistically significant change in FAB scores was found after 6 months of ChEIs treatment. In contrast, a significant reduction in MoCA scores was found between T_0_ and T_1_. The use of ChEIs for 6 months had no impact on depressive symptoms.

**Table 2:** Descriptive statistics and changes in Group 1

Measure	Baseline (T_0_) Mean ± SD	6 months (T_1_) Mean ± SD	Wilcoxon W	p	r_c_
MMSE	20.42 ± 2.278	21.34 ± 3.291	20	0.143	-0.48
MoCA	16.47 ± 3.815	14.58 ± 4.634	75	0.042	0.64
FAB	10.65 ± 3.457	9.765 ± 3.413	88.5	0.11	0.47
HDRS	12.12 ± 7.999	10.47 ± 6.463	62.5	0.247	0.37

Secondly, the effect of ChEIs treatment on cognitive performance and depressive symptoms in mild AD after 9 months in Group 2 was examined (Table 3). Interestingly, the comparison between T_0_ and T_1_ scores showed significant differences in all psychometric tools evaluating cognitive functioning (MMSE, MoCA, and FAB). In particular, the MMSE showed the highest significance, followed by FAB and MoCA. Again, there were no significant differences between T_0_ and T_1_ concerning HDRS scores.

**Table 3:** Descriptive statistics and changes in Group 2

Measure	Baseline (T_0_) Mean ± SD	9 months (T_1_) Mean ± SD	Wilcoxon W	p	r_c_
MMSE	20.47 ± 2.6	17.72 ± 2.241	136	< 0.001	1.0
MoCA	16.14 ± 3.3	12.94 ± 3.431	108	0.007	0.8
FAB	10.93 ± 2.718	8.363 ± 2.957	110	0.005	0.83
HDRS	12.94 ± 7.672	13.56 ± 10.88	61.5	0.755	-0.09

## Discussion

There is a surprising lack of data on the validity of MoCA and FAB in monitoring the effects of drug treatment with ChEIs [11]. Therefore, in the present pilot study the efficacy of MoCA and FAB, compared to the MMSE, in assessing the effect of ChEIs on cognitive decline was evaluated. Furthermore, we also tested whether the HDRS was able to detect any changes in depressive symptoms after taking ChEIs. As a preliminary check, we ensured that Group 1 and Group 2 started with the same cognitive and affective condition.

The comparison between T_0_ and T_1_ in Group 1 showed that there were no significant differences in MMSE scores after 6 months of treatment. When we used also the MoCA and FAB, a clinically relevant difference between T_0_ and T_1_ was found only for the former, but not for the latter; this suggests that MoCA is a more sensitive instrument in detecting a slight worsening in AD patients treated with ChEIs, compared to MMSE and FAB [12, 13]. These results were confirmed by the analysis of the data collected in Group 2, where after 9 months of ChEIs treatment there was a significant decrease in MMSE, MoCA and FAB performance.

The results described above are consistent with those in the literature. For instance, the meta-analysis by Ciesielska et al. found that the MoCA is superior than MMSE as a screening tool for MCI [14]. Furthermore, other authors have shown that MoCA is a very useful psychometric tool not only for MCI, but also for the detection of the early stages of AD [15]. For example, Cecato et al. conducted a cross-sectional study of 136 community-dwelling elderly participants using MMSE and MoCA to evaluate which test was better able to discriminate between healthy controls, MCI and AD. They found that some subtests of the MoCA (i.e., rhino naming, serial 7s, clock drawing, word recall and orientation subtests) differentiated participants with MCI from AD patients [16]. MoCA has also been identified as a well-established tool to track very slight changes in cognition during different treatments, like electroconvulsive therapy [17] and second-generation antidepressants [18], supporting its effective use for monitoring the outcome of clinical protocols. Our study confirms and strengthen these data, also suggesting that MoCA could be a promising tool to better evaluate response to ChEIs, particularly during the first six months of treatment. Despite the limited psychometric profile of our sample, we could hypothesize that MoCA tracked these significant changes because it focuses also on executive deficits, unlike MMSE, and takes into account several different cognitive functions, unlike FAB [19].

One of the main advantages of this pilot study was the comparison of different tests for monitoring the effects of ChEIs in AD. Furthermore, two different follow-up times (i.e., 6 and 9 months) were considered. However, some methodological issues deserve to be mentioned. Firstly, the small sample size certainly limited our inferential power and did not allow to properly estimating ChEIs effects on AD (e.g., the effect on depressive symptoms). Moreover, not all enrolled patients performed a comprehensive neuropsychological assessment at baseline, making it impossible to assess which cognitive aspects were best captured by the MoCA instead of MMSE as a measure of global cognitive impairment. Secondly, the use of a relatively short follow-up is to be considered another limitation of the study (i.e., favorable early cognitive effects may not be maintained in the long term, and late treatment-related side effects may be discovered). Therefore, future studies on this topic should compare an experimental and a control group using a longer follow-up. Finally, although diagnoses of probable AD were carried out by using current criteria, residual confounding (e.g., medical comorbidity, use of other drug classes) should be taken into account.

## Conclusion

The present pilot study suggests the use of MoCA as a preferred global cognition tool for evaluating the effect of treatment with ChEIs in patients with mild AD. Furthermore, the data from the present research suggest the inclusion of MoCA as an outcome measure in future clinical trials aimed at evaluating the efficacy of cognitive enhancers in mild AD patients. Considering the clinical relevance of early detection of AD, and the efficacy of treatment with ChEIs, the use of combined psychometric tools such as MMSE and MoCA for rapid assessment of the degree of cognitive impairment may be a relevant option to facilitate early treatment and improve the quality of life of patients with AD [20, 21].

## Data Availability

The data presented in this study are available on request from the corresponding authors.
